# Structural evolution of water and hydroxyl groups during thermal, mechanical and chemical treatment of high purity natural quartz

**DOI:** 10.1039/d0ra05798c

**Published:** 2020-08-05

**Authors:** Bartłomiej A. Gaweł, Anna Ulvensøen, Katarzyna Łukaszuk, Bjørnar Arstad, Astrid Marie F. Muggerud, Andreas Erbe

**Affiliations:** Department of Materials Science and Engineering, NTNU, Norwegian University of Science and Technology 7491 Trondheim Norway quartz-heat-treatment@the-passivists.org +47 73 594048; The Quartz Corp Strandveien 50 1366 Lysaker Norway; SINTEF Industri Pb. 124 Blindern 0314 Oslo Norway

## Abstract

Fused silica crucibles are commonly used in the fabrication process of solar grade silicon ingots. These crucibles are manufactured from high purity natural quartz sand and as a consequence, their properties are influenced by the presence of water and hydroxyls in the raw quartz. In this work, diffuse reflectance IR, ^1^H magic angle spinning NMR, and Raman spectroscopy were used to investigate the influence of thermal treatment on water and hydroxyl groups in high purity natural quartz sand. Most of the water in dry sand is present in the form of closed inclusions within the quartz grains which were detected in Raman imaging studies, even after thermally treating the samples at 600 °C. Only after heating to 900 °C did this water completely vanish, most likely as a result of rupturing of the inclusions. However, newly formed OH groups, identified as isolated and hydrogen bound OH were observed as products of the reaction between water and quartz. Similarly, liquid water was observed in NMR spectra even after treatment at 600 °C while at temperatures >900 °C, only non-interacting silanol groups were present. The comparison of the temperature dependence of the IR and NMR spectra also yields insight into the assignment of the OH stretching mode region of the IR spectrum in this system. The intensity of water related bands decreases while the intensity of OH bands first increases and then decreases with increasing temperature. The band intensity of Al-rich defects as well as the characteristic feature at 3200 cm^−1^ does not follow the temperature dependence of typical water peaks. It is also shown that leaching the quartz sand in HF solution helps to remove water from inclusions, likely by forming pathways for fluid flow inside the quartz grains. Milling of the samples caused formation of an additional type of hydroxyl group, possibly due to partial amorphisation of the surfaces of the quartz grains surface during the process. The results improve the basis for a knowledge-based processes development for the processing of high purity natural quartz.

## Abstract 2

In dry quartz stable closed liquid micron-size inclusions and newly formed OH groups were observed after thermal treatment.

## Graphical abstract

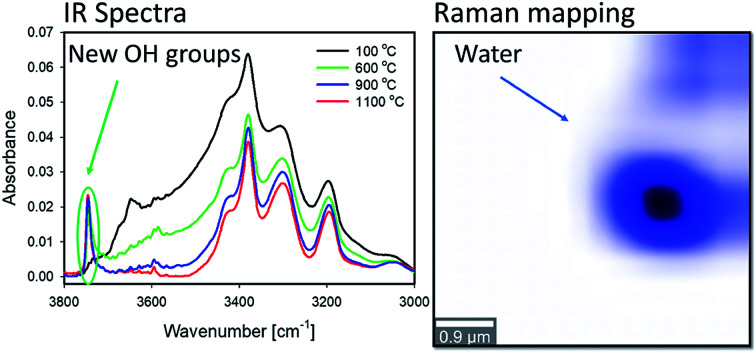

## 1 Introduction

High purity quartz sand is used as the raw material for manufacturing crucibles used in the Czochralski (CZ) production process of high purity crystalline silicon for photovoltaic application. This application demands that the level of contaminants in the quartz sand must be extremely low.^1,2^ Current requirements are typically <20 ppm of metal impurities incorporated in the quartz lattice. Other important impurities include hydroxyl groups and water, at internal and external interfaces, in defects or in inclusions.^3–6^ At the temperatures of the CZ process, the presence of water can lead to hydrolytic weakening by hydrolysis of Si–O bonds, Si–O–Si + H_2_O → 2Si–OH.^7,8^ The consequence of hydrolytic weakening is a lowering of the quartz glass viscosity, rendering crucibles more susceptible to deformation when held at the CZ process temperature of >1500 °C for typical process times of several hundred hours. Hydroxyl groups and molecular water are also responsible for bubble growth in the crucible during the CZ process.^9^ These unwanted features decrease the production yield and reduce the quality of the silicon ingots.^9,10^

Refining of quartz ore into high purity quartz sand comprises several process steps such as mechanical crushing and milling, screening, froth flotation, chemical leaching and thermal treatment.^1^ Flotation and chemical leaching are used to remove most of the metal impurities, which are present as mineral impurities or exist at the quartz grain surface as a result of the milling process. Calcination is used primarily to dry the quartz sand and volatilise any residual organic impurities. Studies of dehydration behaviour in chalcedonic quartz showed that water is mainly located within intergranular spaces and is rapidly removed at 500 °C.^3^ Water removal is mainly associated with water diffusion through incompletely connected grain boundary regions at temperatures above 300 °C.^4^ Other studies emphasised that the presence of water at high temperatures triggers the formation of new hydroxyl groups and dislocations associated with hydrolytic weakening.^8^ However, the characterisation methodology of water and hydroxyl groups in quartz powder is not well established. Thus, the structural evolution of water and hydroxyl groups on natural quartz during different processing steps is not well understood.

Within this scope given by knowledge needs in the field of minerals processing, diffuse reflectance infrared (IR) spectroscopy in the OH stretching mode region, ^1^H magic angle spinning (MAS) nuclear magnetic resonance (NMR) and Raman spectroscopy were used to study the transformation of water in natural α-quartz after heat treatment at different temperatures, different mechanical treatment and different acid treatment. In Section 2, the state of the literature on the correlation between IR absorption features and structure of OH/H_2_O shall be reviewed; a reader only interested in the resulting assignments from this work may skip this section. Section 3 contains experimental details. Section 4.1 shows images and Raman maps of the samples. Spectral features observed in IR and NMR are assigned in detail in Section 4.2, before the effects of milling (Section 4.3), heat treatment (Section 4.4) and acid leaching (Section 4.5) are described. D_2_O exchange experiments (Section 4.6) enable differentiation between external and internal OH. Finally, Section 4.7 discusses the implications of the observed changes in the spectra with different processing parameters.

## 2 OH stretch region in vibrational spectra of hydroxyl groups and water

Since the interpretation of the IR spectra in the region of the OH stretching modes (3000–3800 cm^−1^) of the α-quartz samples is crucial for this paper, we shall briefly discuss the relation between structure and vibrational frequencies, based on experimental, computational and theoretical results. OH stretching modes are expected from bound OH groups or hydroxide ions. This category contains mainly Si–OH groups, but also any contribution from OH groups bound to impurities such as Al–OH. Table 1 summarises some important spectral interpretations associated with relevant species from selected literature. In addition, OH bonds from adsorbed water or water inclusions will also contribute to the OH stretching modes. These are compiled in Table 2.

**Table 1:** Components of OH stretching modes of surface OH groups and in bulk minerals, including assignment to different species according to selected literature, at or around room temperature. The top part of the table contains modes from OH groups and the bottom part those from bulk minerals. Corresponding modes for liquid water are shown in Table 2. Abbreviations: ads – adsorbed; ip – in plane (∥ to surface); op – out of plane (⊥ to surface); ATR-IR – attenuated total reflection IR; DFT (harm.) – density functional theory in harmonic approximation; DFTMD – DFT based molecular dynamics; FA – factor analysis; HB – H-bond; HBA – H-bond acceptor; HBD – H-bond donor; SFG – IR/visible sum frequency generation spectroscopy; TR-IR – transmission IR. References in brackets: compilation of secondary literature

Peak (cm^−1^)	System	Method	Description	Ref.
3824–3688	19/26 Si–OH of am-SiO_2_ + H_2_O_(ads)_	DFT (harm.)	Isolated Si–OH	11
3800–3600	α-Al_2_O_3_(0001) + H_2_O_(ads)_	DFTMD	op Al–OH HBD to H_2_O_(ads)_	12
3780	am-SiO_2_	DFTMD	Isolated Si–OH at low OH coverage	13
≈3750	α-Al_2_O_3_(0001) + H_2_O_(ads)_	DFTMD	Isolated op Al–OH	14
≈3750	Div. SiO_2_	IR, SFG	Isolated Si–OH in air	15
3750	am-SiO_2_	DFTMD	Isolated Si–OH at intermediate OH coverage	13
3747–3737	am-SiO_2_	IR	Isolated Si–OH	(11)
3735	am-SiO_2_	DFTMD	Isolated Si–OH at full OH coverage	13
≈3700	α-Al_2_O_3_(0001) + H_2_O	SFG	Isolated Al–OH	16
3700–3400	am-SiO_2_	DFTMD	Interacting Si–OH at high OH coverage	13
≈3680	am-SiO_2_ + H_2_O	IR, SFG	Isolated Si–OH in H_2_O	15
3610	am-SiO_2_	DFTMD	Interacting Si–OH at high OH coverage	13
≈3600	α-Al_2_O_3_(0001) + H_2_O_(ads)_	DFTMD	Isolated ip Al–OH	14
3600–3000	α-SiO_2_ + H_2_O_(ads)_	DFTMD	Overlapping ip and op Si–OH HBD/HBA with H_2_O_(ads)_	12
3536–3520	am-SiO_2_	IR	Si–OH involved in HB	(11)
3535–3329	7/26 Si–OH of am-SiO_2_ + H_2_O_(ads)_	DFT (harm.)	Si–OH involved in HB	11
3500–3350	α-Al_2_O_3_(0001) + H_2_O_(ads)_	DFTMD	ip Al–OH HBA from H_2_O_(ads)_	12
≈3400	α-Al_2_O_3_(0001) + H_2_O_(ads)_	DFTMD	op Al–OH HBD to H_2_O_(ads)_	14
3670	Porous silica		Internal Si–OH species, grain boundaries	17
3638	Natural muscovite	TR-IR	Structural OH	18
3610	Natural α-SiO_2_	TR-IR	Possibly Al^3+^ related defect	19
3595	Natural and B-doped α-SiO_2_	TR-IR	OH in B^3+^ related defect	19
3585	Natural α-SiO_2_	TR-IR	OH in Al^3+^ related defect or OH groups in dislocations	8 and 19
3511	Natural α-SiO_2_	TR-IR	OH in Li^+^ related defect	19
3486	Natural α-SiO_2_	TR-IR	OH in H^+^ related defect	19
3470	Natural α-SiO_2_	TR-IR	OH in H^+^ related defect	19
3430	Natural and Al- doped α-SiO_2_	TR-IR	OH in Al^3+^ related defect	19
3378	Natural and Al- doped α-SiO_2_	TR-IR	OH in Al^3+^ related defect	19
3313	Natural and Al- doped α-SiO_2_	TR-IR	OH in Al^3+^ related defect	19
3311	Natural muscovite	TR-IR	Structural OH	18
3200	Natural α-SiO_2_	TR-IR	Si–O overtone	19
3146	Natural muscovite	TR-IR	Structural OH	18
3035	Natural muscovite	TR-IR	Structural OH	18

**Table 2:** Components of OH stretching modes of H_2_O and assignment to different species or modes according to selected literature, at or around room temperature. Abbreviations and notation see Table 1

Peak (cm^−1^)	System	Method	Description	Ref.
3756	H_2_O (gas)	IR, Raman	Antisym. str. (H_2_O)	(20)
3730	H_2_O/D_2_O (liq.)	ATR-IR, FA	*ν*_3_ + *ν*_L2_ (H_2_O)	21
3657	H_2_O (gas)	IR, Raman	Sym. str. (H_2_O)	(20)
3640	[Ge(100)–H] + H_2_O	ATR-IR	Non-H bound H_2_O on hydrophobic surface	22 and 23
3629	H_2_O (liq.)	ATR-IR, Gauss fit	“Free” OH	24
3620	H_2_O/D_2_O (liq.)	ATR-IR, FA	*ν*_1_ + *ν*_L2_ (H_2_O)	21
3600–3300	α-SiO_2_(0001) + H_2_O_(ads)_	DFTMD	H_2_O_(ads)_ HBD to ip Si–OH	12
3600–3200	α-Al_2_O_3_(0001) + H_2_O_(ads)_	DFTMD	H_2_O_(ads)_ HBA from op Al–OH	12
3584	H_2_O (liq.)	TR-IR + thermodynamics	H_2_O, coord. numb. <3	25
3535	H_2_O (liq.)	ATR-IR, Gauss fit	OH “bond ordering” 0, 1	24
3528	H_2_O/D_2_O (liq.)	ATR-IR, FA	*ν*_3_ + *ν*_T2_ (H_2_O)	21
3475	H_2_O (liq.)	Electr. struct. calc. + MD	Collective OH str.	20
3462	H_2_O (liq.)	TR-IR + thermodynamics	H_2_O, coord. Numb. ≈3	25
≈3450–3400	α-SiO_2_(0001)/am-SiO_2_ + H_2_O (liq.)	SFG	“Liquid-like” H_2_O_(ads)_	15 and 26
≈3450–3430	α-Al_2_O_3_(0001) + H_2_O	SFG	“Liquid-like” H_2_O_(ads)_	16
3390	H_2_O (liq.)	ATR-IR, Gauss fit	OH “bond ordering” 2	24
3389	H_2_O/D_2_O (liq.)	ATR-IR, FA	*ν*_3_ (H_2_O)	21
3375	H_2_O (liq.)	Electr. struct. calc. + MD	Collective OH str.	20
3350	[Ge(100)–OH] + H_2_O	ATR-IR + transition model	H_2_O_(ads)_ in contact with OH and bulk water	23
3304	H_2_O (liq.)	TR-IR + thermodynamics	Tetrahedrally coordinated H_2_O	25
≈3300	α-SiO_2_(0001) + H_2_O_(ads)_	DFTMD	H_2_O_(ads)_ HBD to ip Si–OH (“liquid-like”)	27
3300–3000	α-SiO_2_(0001) + H_2_O_(ads)_	DFTMD	H_2_O_(ads)_ HBA from op Si–OH	12
3300–3000	α-Al_2_O_3_(0001) + H_2_O_(ads)_	DFTMD	H_2_O_(ads)_ HBD to ip Al–OH	12
3250	H_2_O (liq.)	Electr. struct. calc. + MD	Collective OH str.	20
3246	H_2_O (bulk)	ATR-IR, Gauss fit	OH “bond ordering” 3	24
3240	[Ge(100)–OH] + H_2_O	ATR-IR + transition model	H_2_O_(ads)_ in contact with OH and bulk water	23
3222	H_2_O/D_2_O (bulk)	ATR-IR, FA	*ν*_1_ (H_2_O)	21
≈3200	α-Al_2_O_3_(0001) + H_2_O	SFG	“Ice-like” H_2_O_(ads)_	16
≈3200–3100	α-SiO_2_(0001)/am-SiO_2_ + H_2_O (liq.)	SFG	“Ice-like” H_2_O_(ads)_	15 and 26
≈3160	α-SiO_2_(0001) + H_2_O_(ads)_	DFTMD	H_2_O_(ads)_ HBA from op Si–OH (“ice-like”)	27
3134	H_2_O (bulk)	ATR-IR, Gauss fit	OH “bond ordering” 3′	24
3079	H_2_O/D_2_O (bulk)	ATR-IR, FA	*ν*_1_ − *ν*_T2_ (H_2_O)	21
3045	H_2_O (bulk)	ATR-IR, Gauss fit	OH “bond ordering” 4	24

Vibrational modes are affected by hydrogen bonds (HBs). Harmonic DFT calculations of 26 different Si–OH groups on amorphous “fused silica” (am-SiO_2_) in the presence of adsorbed water showed a gap of *ca.* 150 cm^−1^ in between frequencies of populations involved in H-bonds (3535–3329 cm^−1^) and those not involved in H-bonds (3824–3688 cm^−1^).^11^ Varying the OH surface coverage on amorphous SiO_2_ models showed, however, absorption in the intermediate region around 3600 cm^−1^, attributed to interacting silanols.^13^ H-bound species show up to more than one order of magnitude higher absorption coefficients than non-H-bound Si–OH.^11^ The absorption coefficient of the latter population is also more homogeneous. The high absorption coefficients, in particular from Si–OH groups which are HB-donors only,^11^ are expected to dominate measurements. Harmonic frequencies in general may not quantitatively reproduce experimental results. They are, however, likely reproduce important trends.

Very detailed insight is available from DFT-based molecular dynamics (MD) simulations of adsorbed water on OH-terminated α-SiO_2_(0001) and α-Al_2_O_3_(0001),^12,14,27^ and also for models for amorphous surfaces.^13^ A remarkable result from the comparison of the two systems is that the nature of the H-bond connecting adsorbed water and surface OH is reversed between the two oxide surfaces. As a consequence, a certain vibrational signature is not a unique characteristic of a certain type of adsorbed water molecule.^12^ A further important result from these computational studies is that the second layer of adsorbed water already gives a spectroscopic signature very close to that of bulk water.^12,22,27^ We shall thus refer to water molecules directly bound to the oxide as “adsorbed water” and further layers as “molecular water”. On the other hand, experimental analysis of water in mesoporous silica indicated differences in spectra up to tens of nm pore diameter.^28^

The interpretation of the stronger H-bound surface hydroxyl species is complicated by the superposition of the absorption from molecular water, for which a number of different interpretations exist. For example, 6 components in the OH stretch region of water have been assigned to 6 differently H-bound species in liquid water.^24^ While it is well recognised that different degrees of H-bonding affect OH stretching positions, a particular complication of the interpretation of the water spectra stems from the fact that molecular water must have two fundamental normal modes in the spectral region in question. To that end, on the basis of a factor analysis of spectra from H_2_O/D_2_O mixtures, the complete OH stretch region was assigned to the water normal modes and combinational modes.^21,29^ Using a singular value decomposition based transition model for the analysis of the electrode potential induced changes of the water spectrum at a Ge(100) electrode near a transition from a hydrophobic to a hydrophilic surface, our group found two main components.^23^

Detailed line shape calculations including coupling between different water molecules point to the importance of collective components in the OH stretching modes; in particular, such models appear as a compromise between the different mutually exclusive views of the OH stretching region of the water spectrum described in the previous paragraph.^20^ A comparison between temperature dependent vibrational spectra and thermodynamic data lead to models with the presence of predominantly two^30^ or three^25^ differently H-bound species while multivariate curve resolution of Raman spectra points to coupling of modes well beyond the first hydration shell.^31^ Strong intermolecular coupling which affects the intramolecular modes by the presence of collective modes is also in line with 2D IR experiments and simulations.^32–34^ The coupling has been interpreted as giving rise to phonon-like modes which propagate to nm length scales.^35^ Even for individual OH oscillators in clusters, the feature at 3400 cm^−1^ is accompanied by a lower frequency, phonon-like mode.^36^ The widely distributed and frequency dependent vibrational relaxation times have been interpreted in the picture of a strong structural heterogeneity of H_2_O.^37^ Using the superposition of cluster spectra leads to a surprisingly good reproduction of the liquid water spectrum.^38^ For bulk water, mapping calculated frequencies with local bond situation may provide insight into the relation between spectroscopic properties and bonding, with limitation.^39^ Many details in this process are just beginning to be unravelled by novel techniques.^40^

We do not set out in this work to unravel the controversies about interpretation of the OH stretching modes. Not surprisingly, many different models reproduce the experimental spectra quite well. For this work, a closer look into the different interpretations is important as differently H-bound components in H_2_O, as used *e.g.* in ref. 24 and 25 can easily change with environment and at an interface. However, the normal modes as used in ref. 21 cannot change with the environment though their frequencies may be slightly modified.

The possible confinement in interfacial systems would lower the length of propagation of phonon-like modes as suggested in ref. 35, consequently affect the absorption frequency and thus explain differences observed between experiments and computations in the extent to how far water vibrational spectra are modified by the presence of an interface.^27,28^ Such considerations need to be kept in mind in our attempts to describe the spectrum of adsorbed and molecular water.

Near interfaces, vibrational spectra often show peaks at ≈3200 cm^−1^, resembling the dominating feature in the spectrum of solid ice.^41^ Because of this similarity, this feature is often referred to as “ice-like”,^15,16,25–27^ which unfortunately sometimes leads to misunderstandings of the actual H-bonding situation of interfacial water. For example, electric fields—intrinsically present across interfaces—also lead to more “ice-like” spectra.^42^ while pressure, and consequently stress, present in minerals shift OH stretching modes typically to lower wavenumbers.^27,43^ On the other hand, confinement of water in pores leads to a breakdown of part of the H-bond network, thus leading to modes at higher frequencies.^44,45^

The peaks of liquid water and adsorbed water strongly involved in H-bonding have also been found to be very broad, both experimentally,^15,16,21,24–26,46^ and computationally.^11,12,14,27^ Different reasons may exist for the observed broadness, such as thermal fluctuations, conformational flexibility or broadening upon H-binding.^11,12,27^

In addition to H_2_O molecules, different forms of the hydrated proton and the hydrated hydroxide ion also contribute to the broad OH stretch peaks of strongly H-bound species with conformational flexibility and the presence of different isomers as shown *e.g.* by spectroscopy from isolated clusters.^47–49^ The unusually low vibrational life times for the hydrated proton may also contribute to the broadness of some features.^50^

## 3 Experimental

### 3.1 Materials

The quartz samples used in this work originated from the Spruce Pine deposit, North Carolina, USA. The highest purity quartz sand used in these experiments was obtained by submitting the mined ore to a series of controlled processing steps including mechanical, chemical and thermal treatments. The samples used in this study, had, originally, 230 μm median diameter, with <10% of particles larger than 400 μm and <10% smaller than 120 μm. In this article, we study the effect of different particle size, acid leaching strength and temperature on the content of water and OH groups in the quartz samples. An overview of important sample parameters is listed in Table 3.

**Table 3:** Samples characteristics. Particle size distribution in μm. See footnotes for explanation of the parameters. The particle size distribution was measured using laser diffraction on a CILAS 1180. The particle size was defined using equivalent spheres: the particle size reported as the diameter of an equivalent sphere having the same volume. Indicated heat treatment was conducted in a laboratory furnace

Sample name	*d*10^a^	*d*50^b^	*d*90^c^	Leaching	Heat treatment
1	120	230	400	High	—
1_100	120	230	400	High	100 °C
1_600	120	230	400	High	600 °C
1_900	120	230	400	High	900 °C
1_1100	120	230	400	High	1100 °C
2	120	230	400	Low	—
Micro 1	2	12	30	High	—
Micro 2	—	12	—	Low	—

a *d*10 – 10% of particles in the cumulative distribution below this diameter.

b *d*50 – median; 50% of the particles below this diameter.

c *d*90 – 10% of particles above this diameter.

The purpose of milling as a mechanical treatment is to reduce the size of the ore to the point where quartz can be recovered from other minerals such as mica, feldspar and garnet which are also present. The samples Micro 1 and Micro 2 were additionally modified using a fine autogenous milling technique to reduce the particle size of the material. Acid leaching as a chemical treatment was performed by using hydrofluoric acid at elevated temperature to efficiently etch away surface impurities. Samples 1 and 2 were obtained under different acid leaching conditions in which the HF/quartz ratio of sample 2 was reduced to 34% of the HF/quartz ratio used for sample 1. The final step of high purity quartz processing is thermal treatment, drying, that removes moisture and improves melting behaviour by alteration of any fluid inclusions, leading to a significant reduction in the bubble count in the silica glass.

The trace element content determined by inductively coupled plasma mass spectrometry (ICP-MS) after dissolution in HF is listed in Table 4. The total trace element content (except hydrogen) was below 20 wt ppm.

**Table 4:** Trace element content (wt ppm) as determined by ICP-MS

Sample	Al	B	Ca	Co	Cr	Cu	Fe	K	Li	Mg	Mn	Na	Ni	Ti
1	13	0.1	0.5	0.01	0.01	0.01	0.2	0.5	0.4	0.1	0.1	0.8	0.01	1.2
2	15	—	1.4	—	—	—	0.5	1.1	0.5	—	—	1.2	—	1.3

### 3.2 Methods

Raw data associated with this study is available online.^51^

#### 3.2.1 Diffuse reflectance Fourier transform IR spectroscopy

IR spectral characterisation of quartz samples was performed using a Vertex 80v spectrometer (Bruker) fitted with a Praying Mantis diffuse reflactance accessory (Harrick). A deuterated triglycine sulphate (DTGS) detector was used. IR spectra were acquired by averaging 100 scans at 4 cm^−1^ spectral resolution. Because of the variation in reflectivity of the samples, raw intensity spectra of the quartz samples were divided by a KBr powder reference raw intensity spectrum and then the maximum was normalized to 1. After normalization, a linear baseline between 4000–3800 cm^−1^ as upper and 3000 cm^−1^ as lower limit was subtracted.

The obtained spectra were converted to absorbance by taking their negative decadic logarithm, and will be displayed in this paper in the region above 3000 cm^−1^. The used processing is a common strategy for data treatment of diffuse reflectance spectra of minerals. It shows good linear response with concentration and, in contrast to the Kubelka–Munk transformation, tends to be less affected by baseline corrections.^52–55^ Linearity with concentration was found in a dilution series in KBr of the samples investigated here.^56^ The spectral region below 3000 cm^−1^ could not be investigated in detail by IR, because of the large absorption of Si–O modes and their overtones.

D_2_O exchange experiments were conducted with similar conditions as standard diffuse reflectance IR measurements. A drop of D_2_O (Sigma-Aldrich, 99% of D) was placed on sample 1_100 and spectra were recorded during drying by argon purging of the cell. For this experiment, an in-house built feed through system was used to enable purging of the Praying Mantis cell inside the evacuated sample compartment of the spectrometer.

Absorbance spectra were fitted using the software package Fityk.^57^ First, a spectrum of sample 1_1100 was fitted using Gaussian peaks. Subsequently, other spectra were fitted using the same parameters as a starting point. The positions of the “sharp” peaks have been read from the graph and allowed to vary slightly in the fitting procedure. Peak positions which are present as shoulders were initially estimated based on literature values. Fitting attempts showed that further broad background peaks needed to be considered, as will be described in Section 4.2. In the final stage of the fitting, peak positions have been fixed and peak width and absorbance were allowed to vary, except for the peaks of liquid water. For those peaks, the peak widths were also fixed.

#### 3.2.2 Raman spectroscopic microscopy

Raman maps were acquired using a confocal Raman microscope (Witec Alpha 300 R) in a backscattering geometry with a spectral resolution of 1 cm^−1^. The spectra were collected over a range of 100 cm^−1^ to 3800 cm^−1^ with an excitation wavelength of 532 nm (frequency-doubled Nd:YAG laser operating at 66 mW power). Illumination and detection were performed through a microscope objective of 50× magnification, numerical aperture of 0.75. The resulting hyperspectral images were analysed using the WiTec Project software.

#### 3.2.3 ^1^H MAS NMR experiments

NMR experiments were carried out using a Bruker Avance III spectrometer operating at a magnetic field of 11.74 T. We used a 4 mm double resonance probe at a MAS rate of 12 kHz. The applied ^1^H resonance frequency at this field was 500 MHz. All NMR experiments were of the single-pulse type, *i.e.*, a recovery time followed by a pulse and finally signal acquisition (free induction decay, FID). For each spectrum a total of 800 FIDs were accumulated using 90° pulses, each of which with 3 μs length, and with a recovery time of 5 s.

Before Fourier transform of the averaged FIDs, zero filling and apodization were applied to improve line shape definitions and signal-to-noise ratio. The apodization was done by multiplying the FIDs with a decaying exponential window function with a processing line broadening (LB) factor of 20 Hz. All NMR spectra were then adjusted by proper signal phasing and baseline corrections.

Due to very weak proton signals, we ran identical experiments as described above but with an empty MAS rotor. This spectrum was then subtracted from the sample spectrum to obtain the final spectrum. The peak from air moisture shows up as a negative peak in the spectra at a chemical shift of ≈0.35 ppm.

## 4 Results and discussion

### 4.1 Raman maps of the OH stretching mode region

**Fig. 1:**
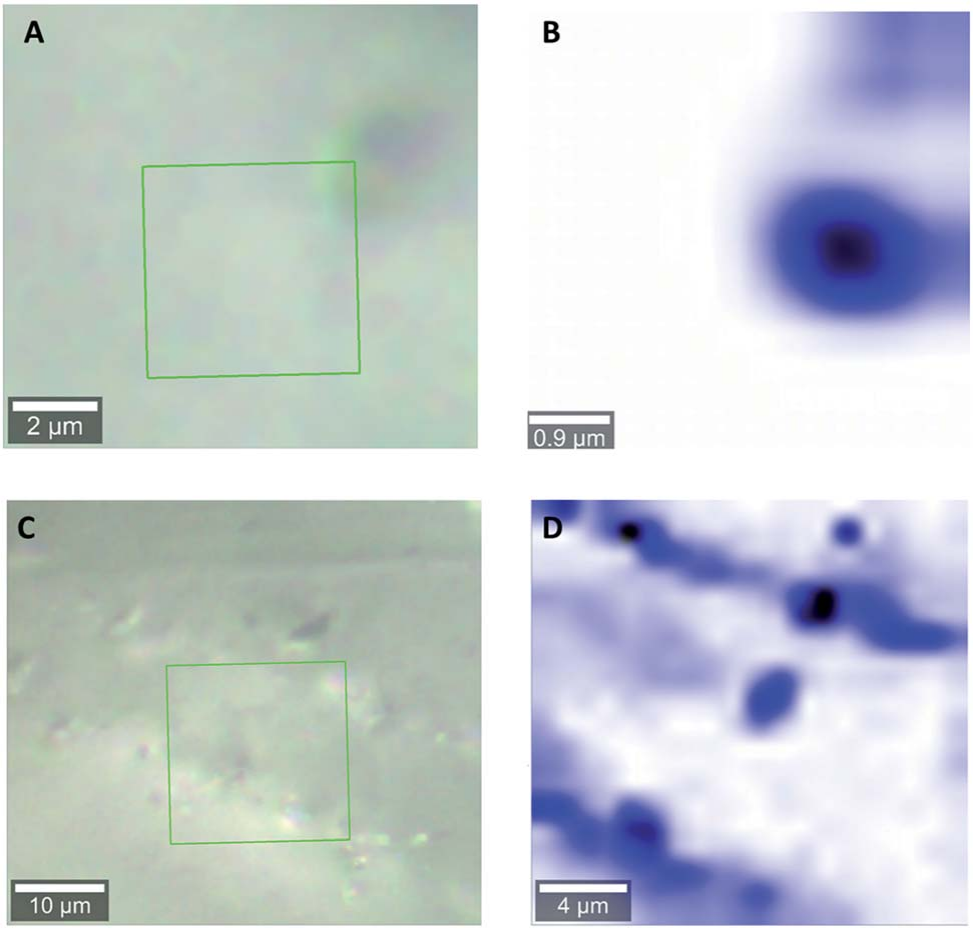
Micrograph of quartz grain of Sample 1 (A) and 2 (C) with corresponding Raman scanning maps (B and D) of the area indicated by a green square in (A) and (C). Raman maps represent false colour images obtained as output of the Raman software's true component analysis. Blue colour represents spectral features of liquid water.

Fig. 1 shows light microscopy images of 1 and 2, and corresponding Raman maps. The Raman maps show the distribution of features associated with liquid water. In the micrographs, there is no clear indication of inclusions. The grain surfaces look smooth, especially in sample 1. However, Raman mapping of the framed area in Fig. 1 indicates clearly the presence of microinclusions with a diameter on the order of 1 μm (Fig. 1B). In sample 2 (Fig. 1C) the detected inclusions have a different shape. Moreover, water was present also in cracks or intercrystallite domains within quartz grains (Fig. 1D).

### 4.2 Band assignment in IR and NMR

**Fig. 2:**
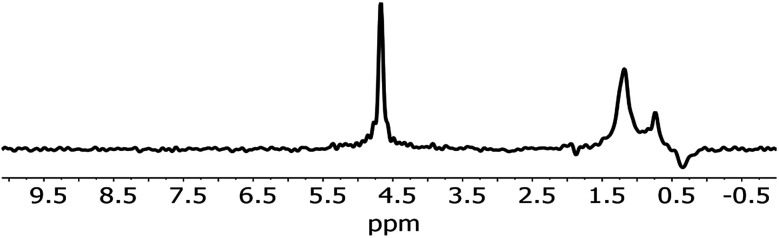
NMR spectrum of 1.

An analysis of the NMR spectrum of a representative sample (Fig. 2) is a good starting point. Three major peaks are observed in the spectra. The peaks at 0.8 and 1.25 ppm likely originate from internal, non-H bound and H-bound hydroxyl groups, respectively.^58^ The sharp peak at 4.7 ppm corresponds to bulk water in inclusions. The negative peak at −0.4 ppm represents water vapour in the empty sample cell. In the spectrum there is no indication of Al–OH related peaks, which are expected to be in the range 2.5–3 ppm.^58^ Overall, as confirmed by modelling,^11,59^ weakly H-bound species show peaks towards lower frequencies, *i.e.* to the right in the spectrum, relative to their H-bound forms. From this NMR spectrum, we can expect at least two different OH species in the IR spectrum.

**Fig. 3:**
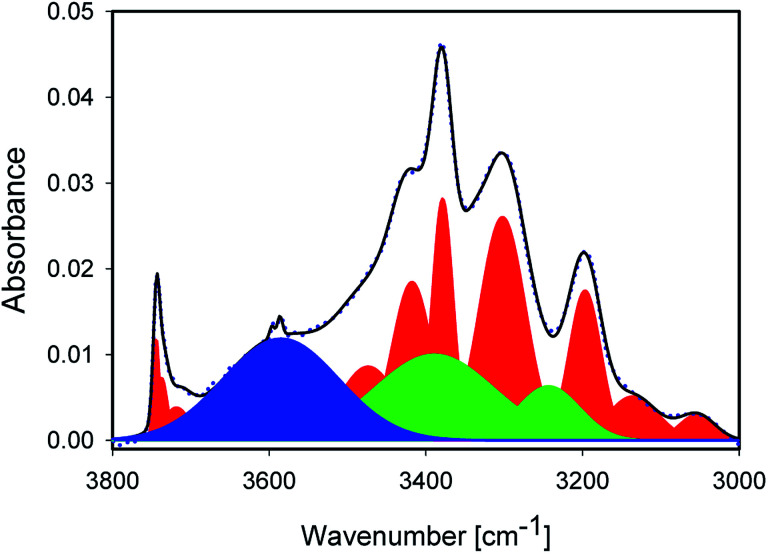
IR spectrum of 1_600 (blue dots) with peak fitting by a superposition of Gaussian peaks (black line). Peaks in red have been assigned to Si–OH or other mineral vibrations. Green peaks represent OH stretching modes of liquid water. The blue peak was assigned to an OH stretching mode of interfacial water.

Fig. 3 shows the IR spectrum of a representative sample, together with a peak fit. Disregarding a few exceptions highlighted below, similar components with different absorbance relative to each other were found in all samples. The spectrum shows significantly more than the 2 components. A detailed comparison of samples is presented later. All IR peak assignments in the range between 3800 and 3000 cm^−1^ are summarised in Table 5, in line with Section 2. The reasoning for the assignment shall be given below, and some of it may become apparent only after looking at the trends observed with sample treatment.

**Table 5:** Peak wavenumber, in cm^−1^, of vibrational modes in the OH stretching mode region with their assignment and corresponding peak, in ppm, in NMR spectra. n.d. – not detected

Peak	NMR	Description	Ref. IR
3750	1.25	Isolated Si–OH	11, 13 and 15
3720	1.25	“Bridged” Si–OH	11 and 15
3680	n.d.	HB Si–OH species at grain boundaries	17
3650	n.d.	HB Si–OH species at grain boundaries, structural OH	17 and 18
3380–3610	n.d.	Adsorbed water, H_2_O_(ads)_ HBD to ip Si–OH or undercoord. H_2_O	12, 13 and 25
3595	n.d.	OH in B^3+^ related defect	19
3585	n.d.	OH in Al^3+^ related defect or OH groups in dislocations	8 and 19
3470	n.d.	Si–OH involved in HB	19
3420	n.d.	OH in Al^3+^ related defect	19
3378	n.d.	OH in Al^3+^ related defect	19
3375	4.7	Liq. H_2_O inclusion	20, 21 and 24
3313	n.d.	OH in Al^3+^ related defect	19
3246	4.7/5.2?^a^	Liq. H_2_O inclusion	20, 21 and 24
3200	n.d.	Natural Si–O overtone?	18 and 19
3146	n.d.	Structural OH in clay-like defects	18
3035	n.d.	Structural OH in clay-like defects	18

a The “?” indicates a tentative assignment.

There is general consensus that the high wavenumber side of the OH stretching mode region shows absorption of OH groups not involved in any H-bonding (Table 2). NMR shows no evidence for the presence of non-H bound Al–OH groups while a clear signal for Si–OH groups is present. We reason thus that the peaks at 3750 and 3720 cm^−1^ originate predominantly from isolated (“free”) and bridged Si–OH groups, respectively.^15,17^

In IR spectra of sample 1_100 (Section 4.4), peaks at 3680 cm^−1^ and 3650 cm^−1^ are also observed, which likely represents internal –OH at grain boundaries.^60^ Alternatively, they may be associated with the presence of aluminosilicate impurities (*e.g.* muscovite).^17,61–63^ This is a region of the spectrum where typically OH donor groups interacting with other OH *via* H-bonds are observed, while acceptors of H-bonds show broad a peak at 3480 cm^−1^.^11^ The weak sharp peak at 3595 cm^−1^ is identified as the B–OH stretching mode,^19^ while the sharp weak band at 3585 cm^−1^ is associated with the hydrogarnet substitution or presence OH groups in dislocations.^64,65^ Both features are commonly present in quartz minerals.^19^ The broad peak at 3480 cm^−1^ indicates OH involved in H-bonding. The peaks at wavenumbers 3420, 3375 and 3313 cm^−1^ are believed to be –OH stretching modes related to the presence of Al-rich defects,^5,8^ although similar peaks are observed for some natural aluminosilicates (*e.g.* muscovite, *cf.*Table 1). These bands are also commonly observed in alumosilicate based synthetic heterogeneous structures.^66^

All of these peaks are sharp which is typical for highly ordered structures. The presence of clay like inclusions in natural quartz is quite common.^61–63^ If the size of their grains is in the nm range, they are difficult to detect using other methods than spectroscopy, especially if their concentration is very low.

The sharp peaks discussed so far are not sufficient to describe the experimental spectra. NMR spectra and Raman maps (Section 4.1) show evidence for the presence of liquid water. Thus, the main peaks at ≈3375 and ≈3250 cm^−1^ needed to be included,^20,21,24,25^ while the minor peaks in the typical water intensity ratios did not affect the fit quality very much. Maxima were manually slightly varied in the early stages of the fitting for these two peaks; the water peaks are marked in green in Fig. 3. It should be stressed that other possibilities would exists to describe the absorbance under the sharp, mineral-related peaks. However, for a consistent interpretation, the inclusion of liquid water is needed, and doing so with peaks fixed to approximately literature maxima appears to us as the most reasonable approach.

With the inclusion of the two main peaks of liquid water, there is still a significant broad background on the high wavenumber side, described with a very broad peak centred at ≈3610 cm^−1^, marked in blue in Fig. 3. The maximum of this peak also varies by about 10 cm^−1^ from sample to sample. From the position, the highest wavenumber mode of the water is located in this region, but the absorbance observed here is significantly higher than in liquid water, so that only the interpretation of weakly H-bound, undercoordinated water would apply. Furthermore, modes of adsorbed, interacting water molecules and confined water are found in this region.^12,13,45^ Because of the decrease observed with increasing treatment temperature (Section 4.4), we consider this mode to be related to adsorbed or confined water. The presence of confinement follows from the features observed in the Raman images (Section 4.1), where water in channel-like structures at grain boundaries was observed.

Finally, the peaks at 3200, 3146 and 3035 cm^−1^ are difficult to assign unambiguously. They may be related to strongly H-bound water. Indeed, the first band is typically attributed to “ice-like”, strongly H-bound water in SFG experiments. Si–O overtones may also play a role in this region. Moreover, in alumosilicates, the structural OH peaks appear this part of the spectrum as well. The absence of a change in temperature of the integrated absorbance for this mode (Section 4.4) makes it likely that this peak is related to overtones or structural OH. On the other hand, the peak is affected by milling (Section 4.3), which would not be immediately obvious, although not impossible, for an Si–O overtone.

The comparison to experimental ^1^H-NMR spectra available here enables a comment on the terminology of “ice-like” water. When looking at the spectra obtained here and comparing them to calculations for liquid water and ice,^59^ we do not find any evidence for the presence of “ice-like” water ‘identified’ by NMR. Because of the H-bond dynamics in ice, if this was present, a significant peak broadening would be expected in the experiments here, at a chemical shift slightly higher than that of liquid water. While the presence of small amounts of “ice-like” water in NMR below the detection limit cannot be excluded, we prefer to abstain from using the term for the IR peak observed here.

When comparing IR to NMR in this work, it is also important to have in mind that the OH stretching mode absorption coefficients for different species can be significantly different.^11,19,67^ Interestingly, evidence exists showing that the absorption coefficient is an extremely sensitive probe for the local structure around an OH bond, while the frequency is also strongly influenced by longer range interactions in the water network.^34^ Here, we shall not attempt to quantify absolutely the different species observed.

The spectral region below 3000 cm^−1^ cannot be investigated in detail by IR, because of the large absorption of Si–O modes and their overtones.

### 4.3 Effect of milling on silanol groups

**Fig. 4:**
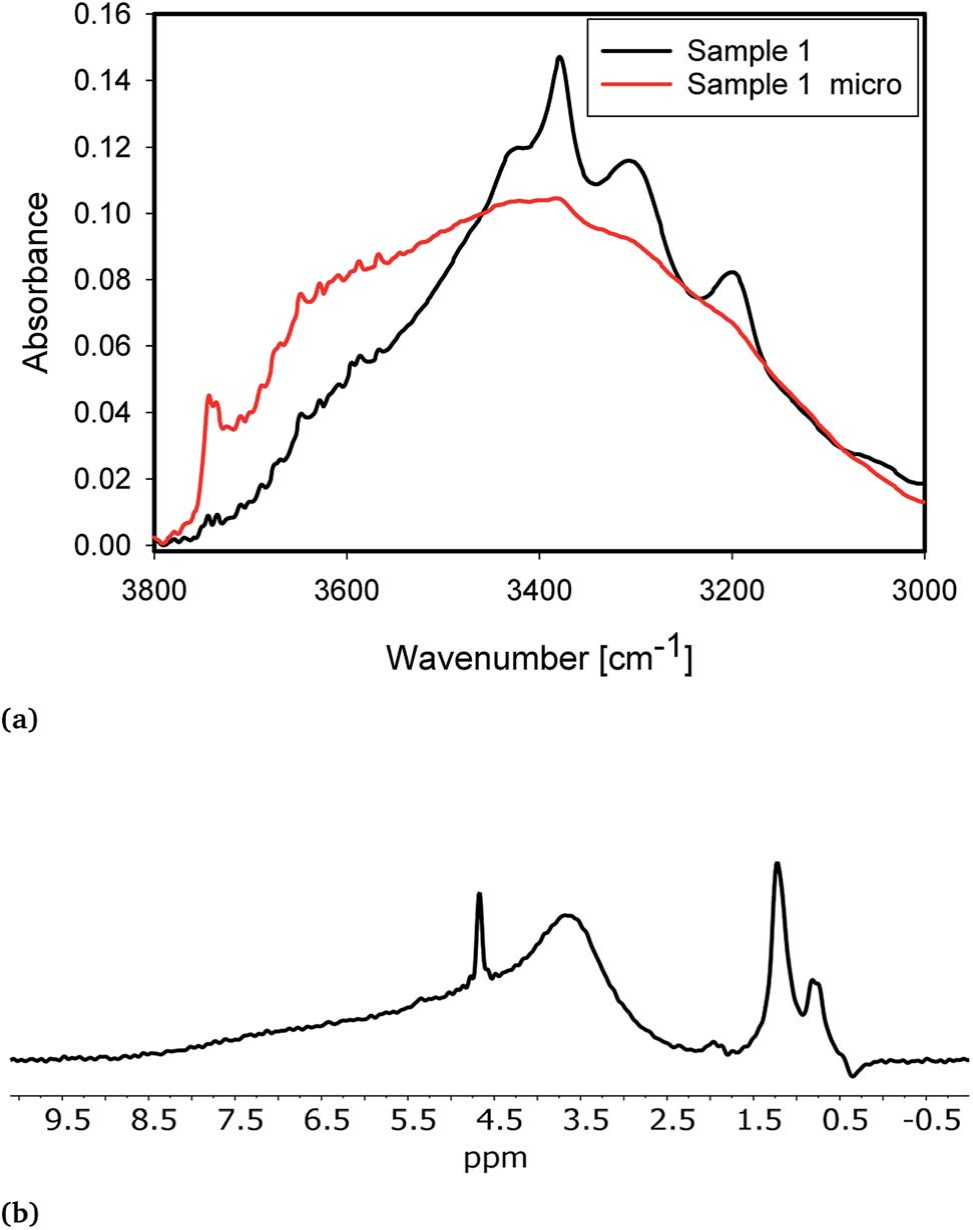
(a) IR spectra of sample 1 (black) and Micro 1 (red). (b) NMR spectra of Micro 1. For comparison, the NMR spectrum of 1 is shown in Fig. 2.

Milling of the samples significantly altered the IR and NMR spectra of the samples (Fig. 4). The samples with 230 μm median particle diameter (*d*50 in Table 3) show much more pronounced Al-related IR peaks at 3420, 3378 and 3313 cm^−1^ compared to fine-milled samples (Micro 1 and Micro 2, Table 3). Moreover, fine-milled samples show significantly increased peaks, in IR at 3750 cm^−1^ and in NMR at ≈1.25 ppm. These peaks correspond to terminal hydroxyl groups. They are significantly more prominent in milled samples because of the significantly larger specific surface area for the (non-porous) milled sample with lower particle diameter.

IR shows the presence of a new peak at 3660 cm^−1^. The NMR spectrum of fine-milled samples shows a new broad peak at ≈3.7 ppm. The additional peaks in both IR and NMR are likely to originate from the same species. Peak positions point to the formation of new H-bound hydroxyl groups of a nearly fully covered surface. From comparison with the peak positions presented from simulations,^13,68^ these groups are likely associated with the formation of new fully hydroxylated surfaces during milling. The IR absorbance of the water bands in both classes of samples is similar. Increased absorbance on the high wavenumber side can be attributed to a change in the internal structure of grains.^8^ In the NMR, the sharp peak at 4.7 ppm from liquid water is slightly weaker, which indicates a mechanical rupture of inclusions and removal of water during milling to a certain extent. This water then transforms into silanol groups covering the surface, as evidenced by the increased absorbance from free and bridged silanols after milling.

### 4.4 Effect of heat treatment

**Fig. 5:**
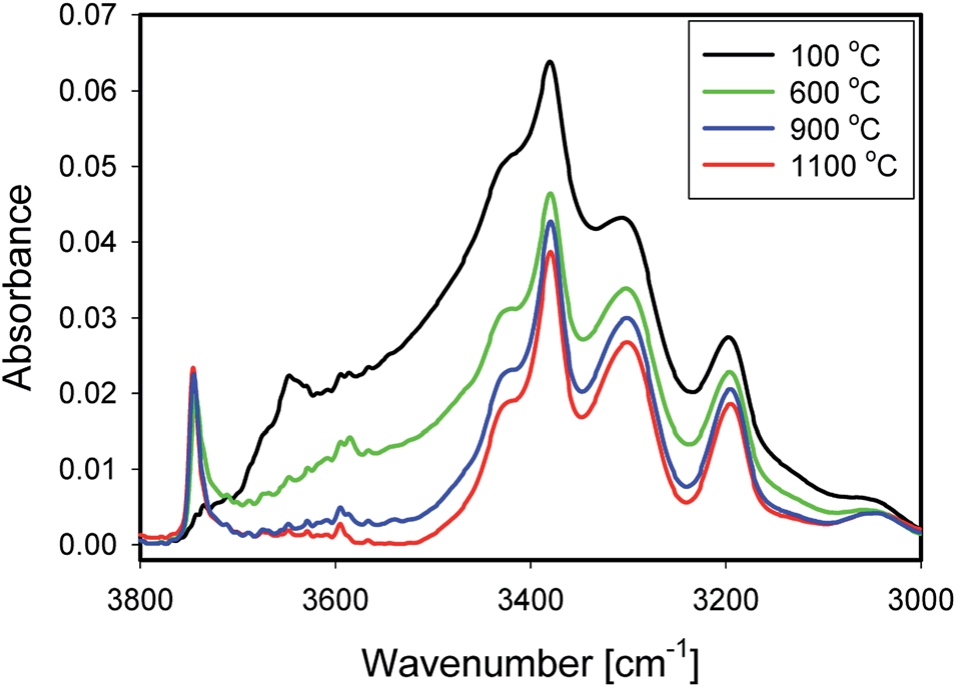
Spectra of 1_100 (black), 1_600 (green), 1_900 (blue) and 1_1100 (red).

**Fig. 6:**
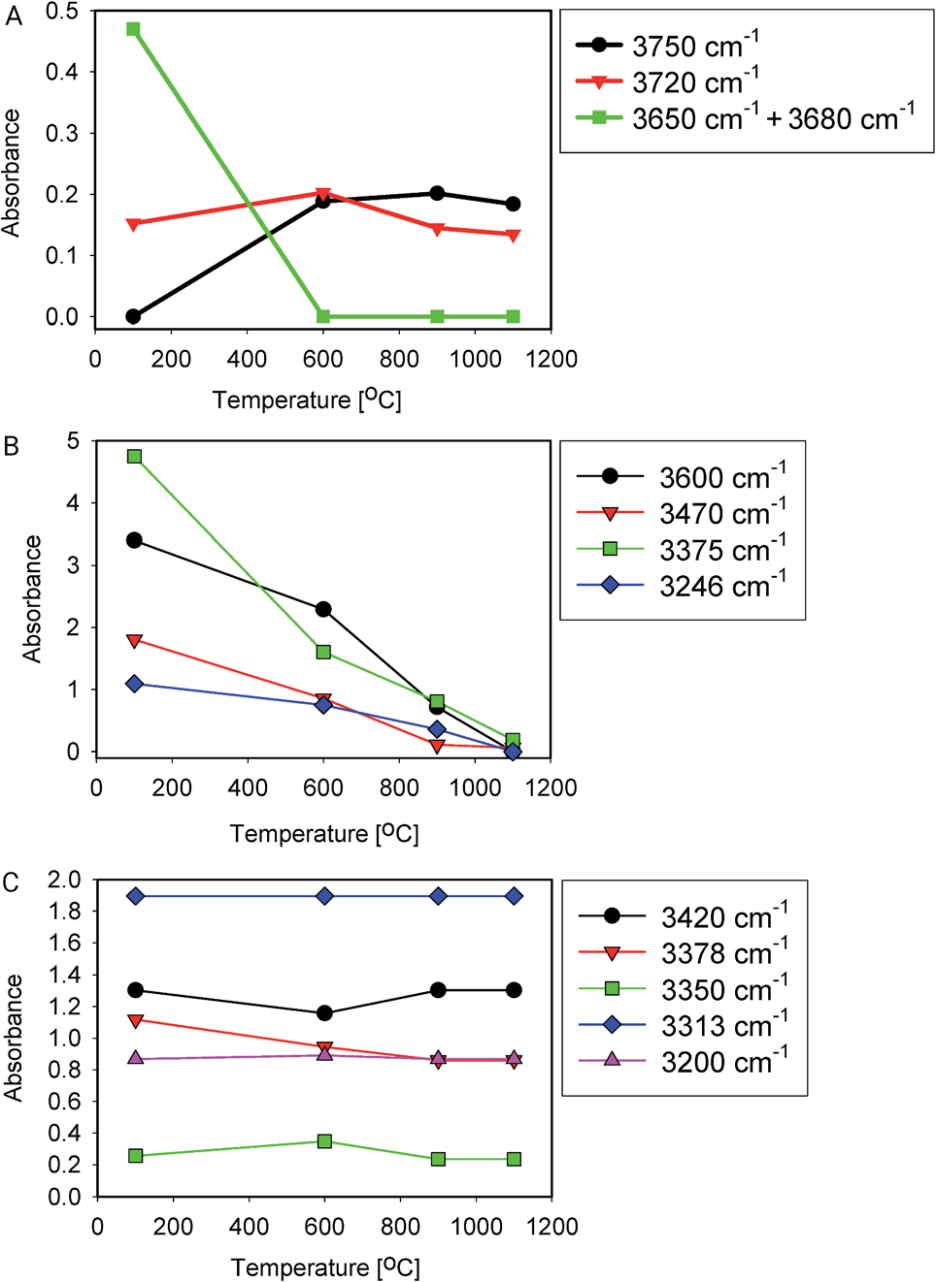
Peak fitting results (area) of heat-treated samples. (A) Si–OH groups, (B) water and bound hydroxyls, (C) Al related –OH groups.

**Fig. 7:**
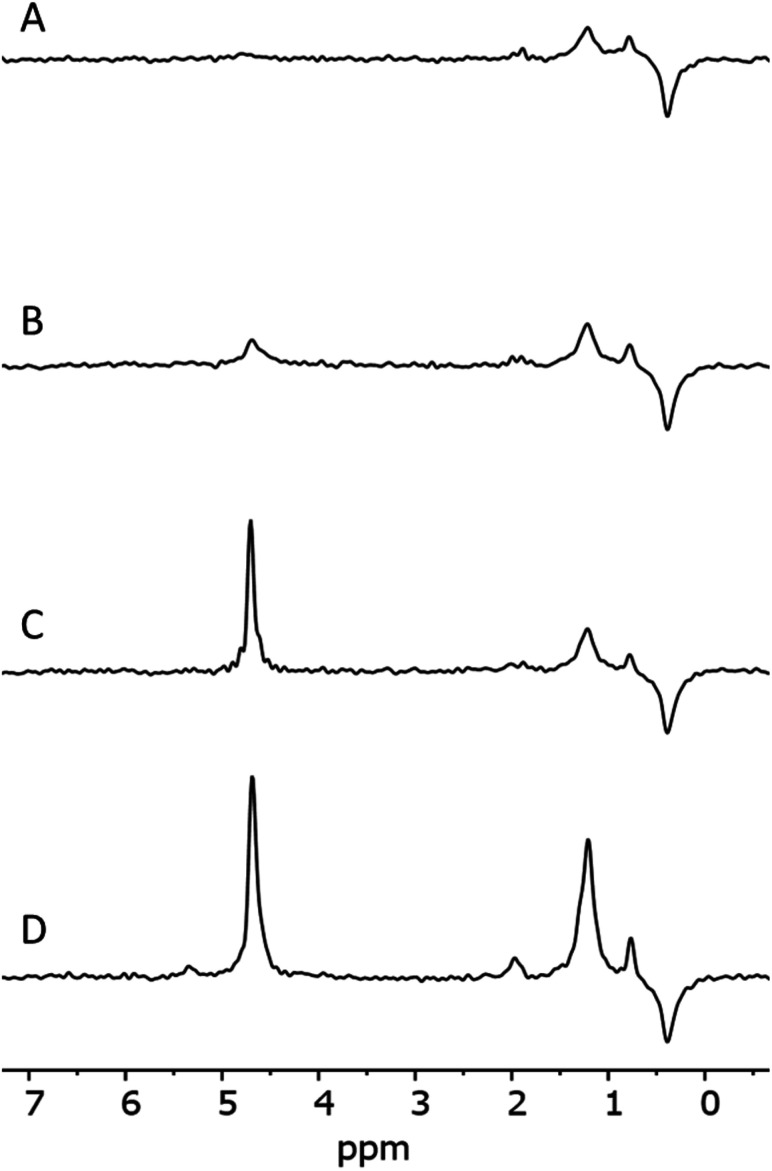
NMR spectra of samples 1_1100 (A), 1_900 (B), 1_600 (C) and 1_100 (D).

Fig. 5 shows IR spectra of samples thermally treated at different temperatures, but measured at room temperature. Peak positions and integrated absorbance obtained from peak fitting are presented in Fig. 6. Corresponding NMR spectra are presented in Fig. 7. IR absorbance between 3400 and 3600 cm^−1^ decreased significantly with an increase of calcination temperature. After treatment at 900 °C, the broad features in the region between 3550 and 3700 cm^−1^ have completely disappeared. The spectra at the highest two temperatures can be described without inclusion of the components from molecular water, though the fit for the spectra from 900 °C are benefit from an inclusion of little residual water. In NMR, the water feature at 4.7 ppm has almost vanished at the same preparation temperature. The peak at 1.25 ppm, assigned to bridged hydroxyls, became significantly less intense at 600 °C but did not reduce at even more elevated temperatures. The decrease in both techniques thus shows a diminishing content of water and bridged hydroxyls (Tables 1, 2 and 5) with increasing temperature of thermal treatment.

In IR, after heat treatment at 600 °C, new peaks at 3750 and 3720 cm^−1^ appeared. These peaks are assigned to formation of non-interacting and H-bound hydroxyls groups. Absorbance of the latter peak decreases after treatment at 900 °C, likely related to condensation reactions.^60^ Al-related peaks are largely preserved during heat treatment.

Moreover, the sharp peak at 3650 cm^−1^ was observed only in one sample, that corresponding to drying at 100 °C. It is likely that this peak is related to the presence of cracks with H-bound Si–OH species;^17^ the same species could explain the NMR peak observed at 2 ppm.

We infer that during heat treatment between 100 and 600 °C, water in most accessible voids and cracks was removed under formation of isolated surface hydroxyl groups. NMR indicates that other hydroxyls are removed at lower temperature compared to water. This observation could be related to the fact that most of the bridged hydroxyls are likely to be at the external surfaces which are relatively easy to evolve, leaving only molecular and adsorbed water in less accessible places remaining. The remaining water is likely present in small inclusions and at grain boundaries deep inside the grains. From the results, this water can be identified by the presence of peaks around 3375 and 3600 cm^−1^.^21,24,25^ Absorbance of both peaks reduces substantially at 600 °C and stabilise after treatment at 900 °C, with an absorbance of ≈25% of that obtained at 100 °C. This residual might be attributed to the presence of alkali defects (Na^+^ and Li^+^). These cations are present in the sample (Table 4), and might affect hydroxyl bond energy and thus lead to separate bands.^69^ The agreement in temperature trends led to the conclusion that the peak at 3600 cm^−1^ originates from adsorbed water acting as H-bond donor to Si–OH, and the peak at ≈3470 cm^−1^ from the Si–OH accepting this H-bond (Table 5).

A discrepancy observed between IR and NMR was the absence of Al–OH peaks in NMR spectra.

Water removal may to some extent be related to the quartz α–β transition at 573 °C because of the reorganisation of the crystal structure and possible formation of new pathways for water removal. However, it is noted that most of the water was removed only after heating the samples to the considerably higher temperature of 900 °C. At 900 °C and above, isolated surface OH groups remain. Isolated OH groups cannot be removed by condensation.

From the viewpoint of assignment of spectral features in IR, it is worth noting that integrated absorbance of some features remain approximately constant with temperature, *i.e.* their change is unsystematic or less than 20% over the whole series (Fig. 6C), while typical water features decrease with temperature (Fig. 6B). Moreover, it is clear from this graph that band at 3470 cm^−1^ is also related to presence of water. It likely represents Si–OH species H-bound to water.^11^ The feature at 3200 cm^−1^, debated in some of the literature,^15,16,27^ does not change with temperature, which is why we do not assign it to surface-bound water here.

### 4.5 Effect of leaching

**Fig. 8:**
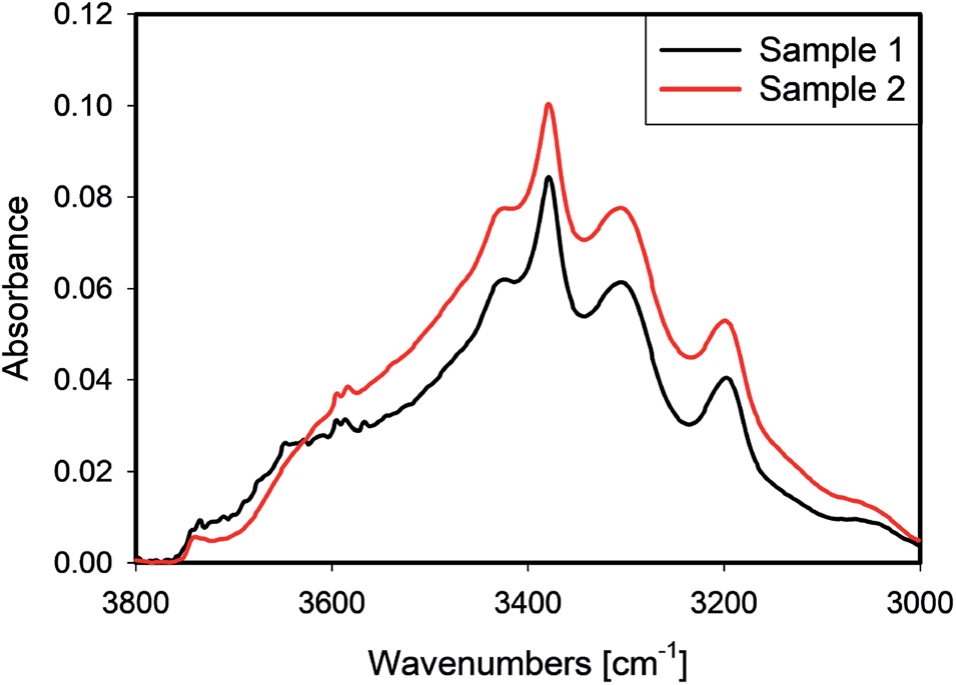
Spectra of 1 and 2 leached under with different acid concentrations conditions respectively.

IR spectra of samples 1 and 2 which were leached with different recipes, but received the same thermal treatment, are presented in Fig. 8. In the spectral region dominated by liquid water, ≈3400 cm^−1^, sample 2 shows higher absorbance, while its absorbance is slightly lower at 3660 cm^−1^. Sample 1 exhibited a sharp peak at ≈3650 cm^−1^ which is often related to the presence of hydroxyls at grain boundaries,^17^ or clay-like impurities.^18^

A comparison of the Raman maps for 1 and 2 (Section 4.1) suggests that more aggressive leaching conditions help to create paths for water transport, which enables more water to be removed at the same heat treatment. Because sample 1 was leached with a higher acid/quartz ratio, one can conclude that the leaching HF not only affects the external surface, but also penetrates deeper into the bulk of the quartz grains. In this process, cracks form and grain boundaries are affected. Moreover, deeper leaching causes more efficient transport of water from any inclusion.

### 4.6 D_2_O exchange

**Fig. 9:**
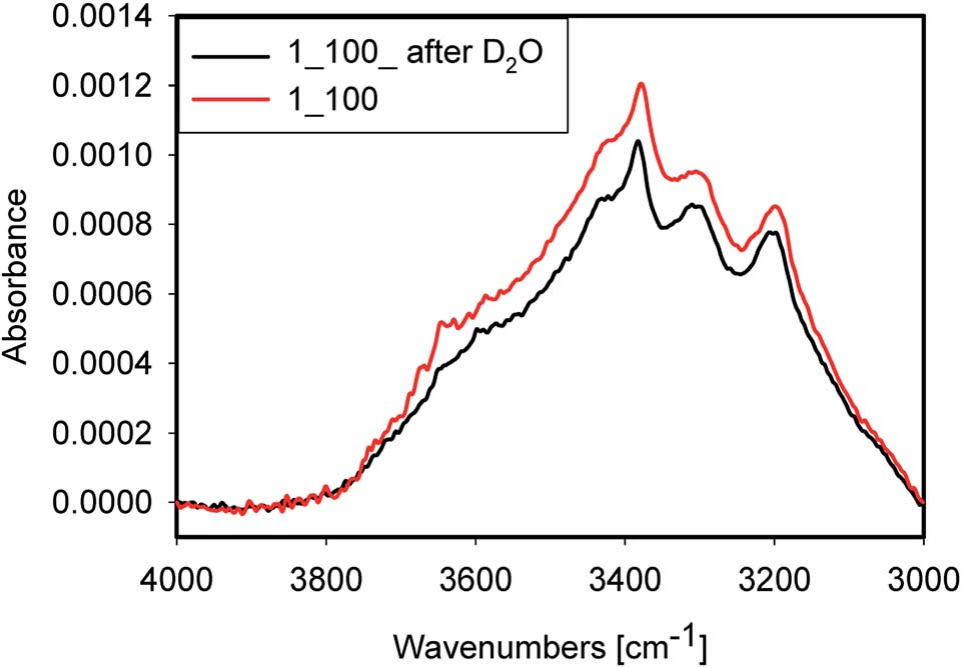
Sample 1_100 before (red) and after soaked with D_2_O (black) at room temperature.

Accessibility of the surface OH groups has been tested by exposing one sample to D_2_O. A spectrum of the original sample and one after evaporation of D_2_O after 3 h of purging with dry argon are shown in Fig. 9. The difference between both spectra was very small. Consequently, the OH groups probed in this experiment are not accessible to D_2_O vapour during the experiment. This result is a strong indication that most of the water is placed inside quartz grains in inclusions and within grain boundaries rather than on the surface of the grain where it would have been accessible to the D_2_O vapour. Nevertheless, a minor decrease of peak intensity at 3650 cm^−1^ after D_2_O treatment was observed, which can be attributed to H-D exchange. Alternatively, this decrease could be due to water removal. Because of strong absorption from Si–O overtones, the OD stretching mode region is not accessible in these samples.

### 4.7 Structural evolution of the water and silanols during processing

**Fig. 10:**
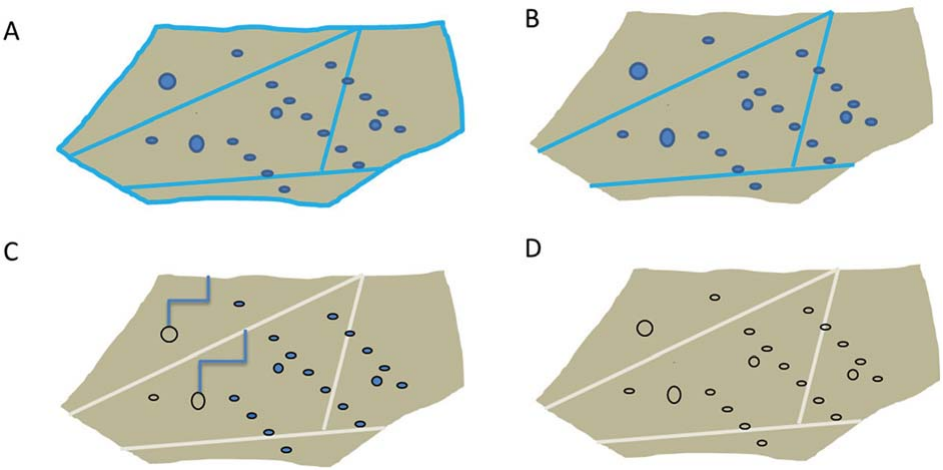
Scheme of drying process of high purity quartz. (A) Sample before drying process, (B) low temperature drying (100 °C), removal of external water, (C) medium temperature treatment (100–600 °C): removal of water from large inclusions and grain boundaries, formation of “free” hydroxyls and (D) high temperature treatment: removal of water from all inclusions, condensation between neighbouring bound hydroxyl groups.

Based on the presented results (Section 4.4), the heat treatment of quartz sand can be divided into 3 stages, as shown in Fig. 10. In the first stage, during drying at around 100 °C only external water is removed (Fig. 10B). In the next step, when the temperature is >100 °C but <600 °C, liquid water from grain boundaries from large inclusions is removed. Only in the third step, above 600 °C when α-quartz is no longer the stable phase, most of the water from the remaining inclusions is removed. Even at 900 °C traces of molecular water are still observed. New hydroxyls which are formed in stage 2 likely form due to reaction between water and SiO_2_; and are discovered to be stable up to 1100 °C. For isolated OH groups to be removed, (i) a high surface mobility is required, so that two groups diffuse until they are close enough to each other to react, or (ii) a homolytic bond breaking of the Si–O bond needs to happen. We hypothesise here, however, that isolated OH groups do not contribute to hydrolytic weakening.

As far as milling as the most important micronisation process is concerned (Section 4.3), large forces during milling likely cause formation of fully hydroxylated surfaces, microcracks and dislocations. The weakest points in grains are often places where grain boundaries or inclusions are present. Therefore, broadening and vanishing of characteristic Al-related bands are expected because of destruction of these kind of local structures. Moreover, the substantial decrease in Al-related features in the spectrum after milling suggests the presence of solid, Al-rich inclusions with submicrometer size (rather than atomic size), randomly distributed defects. These inclusions break during milling and their spectral signature thus becomes less prominent.

The results obtained after leaching in HF (Section 4.5) show that enhanced transport of water along grain boundaries occurs. Also HF leaching decreases the amount of water in the samples, likely by opening alternative transport pathways for the water to exit the grain, and also by generating an osmotic pressure for water outward transport.

In a recent vibrational spectroscopic imaging study, a μm thick interfacial layer between SiO_2_ and H_2_O in a cavity was observed, showing the development of a “diffuse” interface even at room temperature.^70^ It is possible that the transport paths discussed here along grain boundaries and including smaller inclusions occurs according to a mechanism involving similar “swollen” interfacial layers. With the results in this work, it is not possible to confirm or disprove that, however, the shape of the interfaces in Fig. 1D indicates that this is a serious possibility. In a study of water desorption from MCM-41, cavitation phenomena during water removal in pores were found to occur.^71^ The restructuring of the interface observed here suggests that such phenomena may also play a role during heat treatment. The fact that water in porous materials shows deviations from bulk water spectra up to tens of nm of pore sizes,^28^ complicates the IR interpretation.

In summary, the main source of water in high purity quartz is not adsorbed outer surface water, but water in inclusions within the grain. A method to fracture such inclusion by microwaves has recently been developed.^72^ For quartz sand from the deposit used here, however, such a treatment is typically not considered to be necessary.

## 5 Conclusions

Raman mapping showed liquid water inclusions of several μm in size in dry high purity quartz sand, but also “lines” of water, presumably along intercrystallite boundaries. Both were not visible in ordinary micrographs. Results of IR and NMR measurements indicate that most of the water is in the form of closed inclusions. D_2_O exchange experiments show that most of the OH groups are inaccessible from the outside atmosphere. After heating to or above 900 °C, water vanishes from the quartz, probably because of a reaction of the quartz enabling the out transport of the fluid. At these temperatures, only non-hydrogen bound silanol and aluminol groups survive. Aluminium-defect bound OH groups are present in the bulk of the grains, while bridged and free hydroxyl groups are present at their surface. *In situ* experiments may bring deeper insight into the processes at different temperatures.

Milling of samples induces the formation of new forms of hydroxyl groups, most likely at the external grain surface. However the procedure does not cause a significant reduction of water inclusions. Strong leaching improves water removal from inclusions, possibly due to formation of pathways for water diffusion. There are thus different techniques available to remove water from natural quartz. However, the removal of residual, isolated surface OH groups appears to be a significant challenge.

With the presented method combination and the unique sample set, this work is able to provide a scientific base for understanding water inclusions, their modification and their role during different treatments of high purity natural quartz. This work thus presents an essential and significant step forward for knowledge-based processes development from the raw material to the mineral.

## Conflicts of interest

There are no conflicts to declare.

## Supplementary Material
